# 1-Benzyl-3,5-bis­(4-methyl­benzyl­idene)-4-oxopiperidin-1-ium chloride acetic acid monosolvate

**DOI:** 10.1107/S1600536811016138

**Published:** 2011-05-07

**Authors:** Ju-feng Sun, Juan Xing, Jing-tian Han

**Affiliations:** a Binzhou Medical College, Yantai 264003, People’s Republic of China

## Abstract

In the title solvated mol­ecular salt, C_28_H_28_NO^+^·Cl^−^·C_2_H_4_O_2_, the central piperidinium ring of the cation adopts an envelope conformation with the N atom displaced by 0.798 (2) Å from the mean plane of the five C atoms. In the crystal, the components are linked by N—H⋯Cl and O—H⋯Cl hydrogen bonds into trimeric assemblies. C—H⋯Cl and C—H⋯π inter­actions further consolidate the packing.

## Related literature

For background to the use of piperidone derivatives in medicine, see: Dimmock *et al.* (2003 ▶); El-Subbagh *et al.* (2000 ▶); Pati *et al.* (2009 ▶); Das *et al.* (2009 ▶, 2010 ▶). For the synthesis, see: Pati *et al.* (2009 ▶).
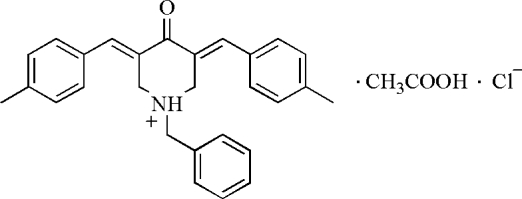


## Experimental

### 

#### Crystal data

C_28_H_28_NO^+^·Cl^−^·C_2_H_4_O_2_
*M* *_r_* = 490.02
Triclinic, 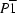

*a* = 7.1488 (5) Å
*b* = 11.2799 (7) Å
*c* = 17.6271 (11) Å
α = 103.181 (6)°
β = 98.087 (6)°
γ = 92.407 (6)°
*V* = 1366.16 (16) Å^3^
*Z* = 2
Mo *K*α radiation
μ = 0.17 mm^−1^
*T* = 288 K
0.61 × 0.54 × 0.52 mm

#### Data collection

Oxford Diffraction Xcalibur Eos Gemini diffractometer
Absorption correction: multi-scan (*CrysAlis PRO*; Oxford Diffraction, 2010 ▶) *T*_min_ = 0.804, *T*_max_ = 1.000
16829 measured reflections
5549 independent reflections
3895 reflections with *I* > 2σ(*I*)
*R*_int_ = 0.029

#### Refinement

*R*[*F* ^2^ > 2σ(*F* ^2^)] = 0.054
*wR*(*F* ^2^) = 0.158
*S* = 1.03
5549 reflections
320 parameters
39 restraints
H-atom parameters constrained
Δρ_max_ = 0.30 e Å^−3^
Δρ_min_ = −0.29 e Å^−3^

### 

Data collection: *CrysAlis PRO* (Oxford Diffraction, 2010 ▶); cell refinement: *CrysAlis PRO*; data reduction: *CrysAlis PRO*; program(s) used to solve structure: *SHELXS97* (Sheldrick, 2008 ▶); program(s) used to refine structure: *SHELXL97* (Sheldrick, 2008 ▶); molecular graphics: *OLEX2* (Dolomanov *et al.*, 2009 ▶); software used to prepare material for publication: *OLEX2*.

## Supplementary Material

Crystal structure: contains datablocks I, global. DOI: 10.1107/S1600536811016138/hb5860sup1.cif

Structure factors: contains datablocks I. DOI: 10.1107/S1600536811016138/hb5860Isup2.hkl

Supplementary material file. DOI: 10.1107/S1600536811016138/hb5860Isup3.cml

Additional supplementary materials:  crystallographic information; 3D view; checkCIF report

## Figures and Tables

**Table 1:** Hydrogen-bond geometry (Å, °) *Cg*3 is the centroid of the C15–C20 ring.

*D*—H⋯*A*	*D*—H	H⋯*A*	*D*⋯*A*	*D*—H⋯*A*
N1—H1⋯Cl1	0.91	2.15	3.0490 (17)	171
O3—H3⋯Cl1	0.82	2.26	3.053 (2)	162
C11—H11*A*⋯Cl1^i^	0.97	2.72	3.602 (2)	151
C25—H25⋯*Cg*3^ii^	0.93	2.85	3.582 (4)	137
C30—H30*C*⋯*Cg*3^iii^	0.97	2.96	3.675 (4)	133

Symmetry codes: (i) 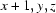; (ii) 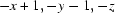; (iii) 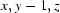.

**Fig. 1.**
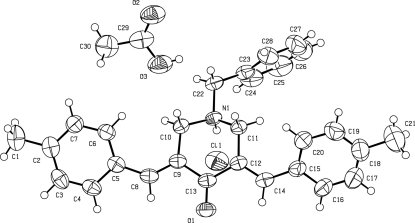
The molecular structure of (I), showing 50% probability displacement ellipsoids.

## supplementary crystallographic information

### Comment

At present, a series of *N*-substituted-3,5-bis(arylidene)-4-piperidone derivatives have been synthesized and proved to be a kind of lead tumor-specific cytotoxin which induces apoptosis and autophagy with multidrug-resistance reverting properties (Pati *et al.* 2009; Das *et al.* 2009; Das *et al.* 2010). These compounds have a marked affinity for thiols in contrast to amino and hydroxyl groups found in nucleic acids (Dimmock *et al.* 2003). Thus development of these compounds as candidate cytotoxics may lead to drugs which are lack of the genotoxic properties present in various antineoplastic agents (El-Subbagh *et al.* 2000). Here, we report the title compound (I), whose IC_50_ (µ*M*) to HL-60 and HSC-2 cells are 59.80 and 105.09 could be used as a basic unit to prepare antineoplastic compounds.

The molecular structure of the title compound (I) is shown in Fig. 1. The hydrogen proton of hydrogen chloride have completely transferred to N1, resulting in the formation of ammonium salt, in which the hydrogen-bonding donors and acceptors reside separately on the cations and anions. In the crystal, weak intermolecular N—H···Cl hydrogen bonds, C—H···Cl hydrogen bonds, O—H···Cl hydrogen bonds and C—H···π stacking interactions contribute to the crystal packing arrangement (Table 1).

### Experimental

The title compound was synthesized according to the literature (Pati *et al.* 2009). Dry hydrogen chloride was continuously bubbled into a solution of *N*-benzyl-4-piperidone (0.005 mol) and *p*-tolualdehyde (0.01 mol) in acetic acid (15 ml) at room temperature. And then the mixture was stirred at room temperature for 12 h. When the produced precipitate was collected, they were added to a solution of aqueous potassium carbonate solution (25%, *w*/*v*). The desired product was obtained after the solid was crystallized by the mixed solvents of ethanol and chloroform (5:1, v/v) in a yield of 74.8%. Yellow blocks of (I) were obtained by slow evaporation of the reacting solution of the title compound in acetic acid.

### Refinement

The H atoms were all located in a different map, but those attached to carbon atoms were repositioned geometrically. The H atoms were initially refined with soft restraints on the bond lengths and angles to regularize their geometry (C—H in the range 0.93–0.98, N—H in the range 0.86–0.89 N—H to 0.86 O—H = 0.82 Å) and *U*_iso_(H) (in the range 1.2–1.5 times *U*_eq_ of the parent atom), after which the positions were refined with riding constraints.

### Crystal data

**Table:**

C_28_H_28_NO^+^·Cl^−^·C_2_H_4_O_2_	*Z* = 2
*M**_r_* = 490.02	*F*(000) = 520
Triclinic, *P*1	*D*_x_ = 1.191 Mg m^−^^3^
*a* = 7.1488 (5) Å	Mo *K*α radiation, λ = 0.7107 Å
*b* = 11.2799 (7) Å	Cell parameters from 6467 reflections
*c* = 17.6271 (11) Å	θ = 3.3–28.9°
α = 103.181 (6)°	µ = 0.17 mm^−^^1^
β = 98.087 (6)°	*T* = 288 K
γ = 92.407 (6)°	Block, yellow
*V* = 1366.16 (16) Å^3^	0.61 × 0.54 × 0.52 mm

### Data collection

**Table:**

Oxford Diffraction Xcalibur Eos Gemini diffractometer	5549 independent reflections
Radiation source: Enhance (Mo) X-ray Source	3895 reflections with *I* > 2σ(*I*)
graphite	*R*_int_ = 0.029
Detector resolution: 16.0355 pixels mm^-1^	θ_max_ = 26.4°, θ_min_ = 3.3°
ω scans	*h* = −8→8
Absorption correction: multi-scan (*CrysAlis PRO*; Oxford Diffraction, 2010)	*k* = −14→14
*T*_min_ = 0.804, *T*_max_ = 1.000	*l* = −22→22
16829 measured reflections	

### Refinement

**Table:**

Refinement on *F*^2^	Primary atom site location: structure-invariant direct methods
Least-squares matrix: full	Secondary atom site location: difference Fourier map
*R*[*F*^2^ > 2σ(*F*^2^)] = 0.054	Hydrogen site location: inferred from neighbouring sites
*wR*(*F*^2^) = 0.158	H-atom parameters constrained
*S* = 1.03	*w* = 1/[σ^2^(*F*_o_^2^) + (0.0749*P*)^2^ + 0.3122*P*] where *P* = (*F*_o_^2^ + 2*F*_c_^2^)/3
5549 reflections	(Δ/σ)_max_ < 0.001
320 parameters	Δρ_max_ = 0.30 e Å^−^^3^
39 restraints	Δρ_min_ = −0.29 e Å^−^^3^

### Special details

**Table:**

Geometry. All e.s.d.'s (except the e.s.d. in the dihedral angle between two l.s. planes) are estimated using the full covariance matrix. The cell e.s.d.'s are taken into account individually in the estimation of e.s.d.'s in distances, angles and torsion angles; correlations between e.s.d.'s in cell parameters are only used when they are defined by crystal symmetry. An approximate (isotropic) treatment of cell e.s.d.'s is used for estimating e.s.d.'s involving l.s. planes.
Refinement. Refinement of *F*^2^ against ALL reflections. The weighted *R*-factor *wR* and goodness of fit *S* are based on *F*^2^, conventional *R*-factors *R* are based on *F*, with *F* set to zero for negative *F*^2^. The threshold expression of *F*^2^ > σ(*F*^2^) is used only for calculating *R*-factors(gt) *etc*. and is not relevant to the choice of reflections for refinement. *R*-factors based on *F*^2^ are statistically about twice as large as those based on *F*, and *R*- factors based on ALL data will be even larger.

### Fractional atomic coordinates and isotropic or equivalent isotropic displacement parameters (Å^2^)

**Table:**

	*x*	*y*	*z*	*U*_iso_*/*U*_eq_	
Cl1	0.08804 (8)	0.75303 (7)	0.28289 (4)	0.0748 (3)	
N1	0.5035 (2)	0.71709 (14)	0.26944 (10)	0.0393 (4)	
H1	0.3839	0.7342	0.2781	0.047*	
C9	0.3879 (3)	0.65470 (19)	0.12495 (12)	0.0419 (5)	
O1	0.2783 (2)	0.82076 (15)	0.07466 (10)	0.0620 (5)	
C13	0.3747 (3)	0.7863 (2)	0.12748 (13)	0.0449 (5)	
C12	0.4888 (3)	0.87647 (18)	0.19513 (12)	0.0416 (5)	
C11	0.6027 (3)	0.82996 (18)	0.25930 (12)	0.0426 (5)	
H11A	0.7271	0.8123	0.2457	0.051*	
H11B	0.6192	0.8920	0.3084	0.051*	
C10	0.4892 (3)	0.61825 (18)	0.19628 (12)	0.0437 (5)	
H10A	0.4218	0.5462	0.2035	0.052*	
H10B	0.6156	0.5973	0.1871	0.052*	
C5	0.3050 (3)	0.4412 (2)	0.03506 (12)	0.0469 (5)	
C8	0.3123 (3)	0.5739 (2)	0.05816 (13)	0.0468 (5)	
H8	0.2536	0.6094	0.0193	0.056*	
C14	0.4858 (3)	0.9947 (2)	0.19560 (13)	0.0498 (5)	
H14	0.3996	1.0132	0.1561	0.060*	
C4	0.1820 (3)	0.3827 (2)	−0.03376 (13)	0.0538 (6)	
H4	0.1132	0.4299	−0.0629	0.065*	
C22	0.5994 (3)	0.6717 (2)	0.33817 (12)	0.0502 (5)	
H22A	0.5301	0.5970	0.3402	0.060*	
H22B	0.7261	0.6518	0.3288	0.060*	
C23	0.6138 (3)	0.7602 (2)	0.41694 (13)	0.0522 (6)	
C6	0.4122 (3)	0.3656 (2)	0.07377 (14)	0.0543 (6)	
H6	0.5010	0.4004	0.1180	0.065*	
C15	0.5982 (4)	1.0990 (2)	0.24915 (13)	0.0557 (6)	
C2	0.2600 (3)	0.1835 (2)	−0.01829 (14)	0.0571 (6)	
C28	0.7816 (4)	0.8286 (3)	0.45080 (15)	0.0677 (7)	
H28	0.8854	0.8218	0.4240	0.081*	
C3	0.1601 (3)	0.2573 (2)	−0.05945 (14)	0.0591 (6)	
H3A	0.0767	0.2218	−0.1052	0.071*	
C7	0.3887 (4)	0.2404 (2)	0.04754 (14)	0.0583 (6)	
H7	0.4615	0.1927	0.0749	0.070*	
C24	0.4610 (4)	0.7721 (3)	0.45812 (16)	0.0724 (8)	
H24	0.3459	0.7279	0.4360	0.087*	
C20	0.7861 (4)	1.0932 (2)	0.28108 (15)	0.0649 (7)	
H20	0.8430	1.0198	0.2699	0.078*	
C19	0.8889 (5)	1.1962 (3)	0.32947 (17)	0.0867 (9)	
H19	1.0147	1.1917	0.3502	0.104*	
C1	0.2305 (5)	0.0466 (2)	−0.04400 (18)	0.0793 (8)	
H1A	0.1202	0.0239	−0.0836	0.119*	
H1B	0.2132	0.0140	0.0005	0.119*	
H1C	0.3394	0.0145	−0.0653	0.119*	
C16	0.5197 (5)	1.2108 (2)	0.26610 (17)	0.0773 (8)	
H16	0.3962	1.2174	0.2434	0.093*	
C27	0.7962 (5)	0.9078 (3)	0.52492 (17)	0.0869 (10)	
H27	0.9094	0.9542	0.5472	0.104*	
C18	0.8059 (6)	1.3060 (3)	0.34730 (18)	0.0944 (10)	
C25	0.4813 (6)	0.8509 (4)	0.53299 (19)	0.0936 (11)	
H25	0.3800	0.8573	0.5611	0.112*	
C26	0.6465 (7)	0.9179 (3)	0.56492 (18)	0.0940 (11)	
H26	0.6576	0.9710	0.6144	0.113*	
C17	0.6200 (6)	1.3104 (3)	0.3151 (2)	0.0997 (11)	
H17	0.5620	1.3831	0.3273	0.120*	
O2	0.1938 (3)	0.4138 (2)	0.24132 (14)	0.0879 (6)	
O3	−0.0627 (3)	0.4934 (2)	0.19550 (14)	0.0900 (7)	
H3	−0.0103	0.5553	0.2265	0.135*	
C29	0.0411 (4)	0.4005 (3)	0.19967 (18)	0.0713 (8)	
C30	−0.0443 (5)	0.2837 (3)	0.1471 (2)	0.0894 (9)	
H30A	−0.0568	0.2901	0.0932	0.134*	
H30B	−0.1671	0.2656	0.1596	0.134*	
H30C	0.0355	0.2195	0.1541	0.134*	
C21	0.9236 (8)	1.4166 (4)	0.4024 (3)	0.1380 (15)	
H21A	1.0508	1.3954	0.4156	0.207*	
H21B	0.8685	1.4410	0.4496	0.207*	
H21C	0.9255	1.4828	0.3765	0.207*	

### Atomic displacement parameters (Å^2^)

**Table:**

	*U*^11^	*U*^22^	*U*^33^	*U*^12^	*U*^13^	*U*^23^
Cl1	0.0409 (4)	0.1028 (6)	0.0838 (5)	0.0246 (3)	0.0192 (3)	0.0190 (4)
N1	0.0316 (8)	0.0463 (9)	0.0458 (9)	0.0111 (7)	0.0108 (7)	0.0182 (7)
C9	0.0290 (10)	0.0530 (12)	0.0471 (12)	0.0065 (8)	0.0079 (8)	0.0170 (9)
O1	0.0554 (10)	0.0650 (10)	0.0671 (11)	0.0056 (8)	−0.0080 (8)	0.0292 (8)
C13	0.0328 (10)	0.0563 (12)	0.0514 (12)	0.0071 (9)	0.0078 (9)	0.0233 (10)
C12	0.0329 (10)	0.0491 (11)	0.0488 (12)	0.0089 (8)	0.0123 (8)	0.0193 (9)
C11	0.0359 (11)	0.0452 (11)	0.0491 (12)	0.0067 (8)	0.0065 (9)	0.0155 (9)
C10	0.0402 (11)	0.0442 (11)	0.0488 (12)	0.0089 (9)	0.0074 (9)	0.0138 (9)
C5	0.0365 (11)	0.0590 (13)	0.0461 (12)	0.0046 (9)	0.0107 (9)	0.0114 (10)
C8	0.0339 (11)	0.0616 (13)	0.0488 (12)	0.0076 (9)	0.0064 (9)	0.0207 (10)
C14	0.0503 (13)	0.0547 (13)	0.0508 (12)	0.0127 (10)	0.0088 (10)	0.0232 (10)
C4	0.0383 (12)	0.0723 (16)	0.0496 (13)	0.0066 (11)	0.0051 (9)	0.0123 (11)
C22	0.0541 (13)	0.0519 (12)	0.0509 (13)	0.0162 (10)	0.0073 (10)	0.0235 (10)
C23	0.0595 (14)	0.0587 (13)	0.0469 (12)	0.0178 (11)	0.0111 (10)	0.0258 (10)
C6	0.0517 (14)	0.0603 (14)	0.0477 (12)	0.0081 (11)	0.0014 (10)	0.0093 (10)
C15	0.0758 (16)	0.0502 (12)	0.0460 (12)	0.0097 (11)	0.0086 (11)	0.0211 (10)
C2	0.0551 (14)	0.0621 (14)	0.0543 (14)	−0.0026 (11)	0.0239 (11)	0.0060 (11)
C28	0.0702 (18)	0.0816 (18)	0.0539 (15)	0.0056 (14)	0.0070 (13)	0.0229 (13)
C3	0.0421 (13)	0.0734 (16)	0.0532 (13)	−0.0062 (11)	0.0077 (10)	−0.0008 (12)
C7	0.0685 (16)	0.0557 (14)	0.0511 (13)	0.0095 (12)	0.0099 (11)	0.0123 (11)
C24	0.0694 (18)	0.098 (2)	0.0609 (16)	0.0197 (15)	0.0209 (13)	0.0324 (15)
C20	0.0783 (17)	0.0536 (13)	0.0644 (15)	−0.0023 (12)	0.0025 (13)	0.0235 (11)
C19	0.107 (2)	0.0785 (17)	0.0695 (17)	−0.0101 (15)	−0.0170 (15)	0.0289 (14)
C1	0.095 (2)	0.0636 (17)	0.0759 (18)	−0.0121 (15)	0.0294 (16)	0.0030 (14)
C16	0.114 (2)	0.0523 (14)	0.0656 (16)	0.0224 (14)	0.0013 (15)	0.0184 (12)
C27	0.113 (3)	0.086 (2)	0.0567 (17)	−0.0024 (19)	−0.0056 (17)	0.0195 (15)
C18	0.150 (3)	0.0600 (15)	0.0632 (17)	−0.0108 (17)	−0.0071 (18)	0.0127 (13)
C25	0.112 (3)	0.125 (3)	0.0609 (18)	0.046 (2)	0.0394 (18)	0.0355 (19)
C26	0.144 (4)	0.092 (2)	0.0497 (17)	0.034 (2)	0.017 (2)	0.0177 (15)
C17	0.149 (3)	0.0584 (16)	0.085 (2)	0.0182 (17)	−0.004 (2)	0.0138 (14)
O2	0.0612 (13)	0.1007 (15)	0.1090 (16)	0.0147 (11)	0.0039 (12)	0.0436 (13)
O3	0.0621 (13)	0.1004 (16)	0.1116 (18)	0.0188 (12)	−0.0014 (11)	0.0401 (14)
C29	0.0529 (16)	0.095 (2)	0.0828 (19)	0.0131 (15)	0.0192 (14)	0.0479 (17)
C30	0.085 (2)	0.092 (2)	0.096 (2)	−0.0020 (18)	0.0156 (18)	0.0338 (19)
C21	0.181 (3)	0.100 (2)	0.105 (2)	−0.017 (2)	−0.024 (2)	0.001 (2)

### Geometric parameters (Å, °)

**Table:**

N1—H1	0.9100	C2—C1	1.504 (4)
N1—C11	1.490 (3)	C28—H28	0.9300
N1—C10	1.488 (3)	C28—C27	1.392 (4)
N1—C22	1.510 (3)	C3—H3A	0.9300
C9—C13	1.482 (3)	C7—H7	0.9300
C9—C10	1.510 (3)	C24—H24	0.9300
C9—C8	1.341 (3)	C24—C25	1.398 (4)
O1—C13	1.225 (2)	C20—H20	0.9300
C13—C12	1.492 (3)	C20—C19	1.385 (4)
C12—C11	1.505 (3)	C19—H19	0.9300
C12—C14	1.333 (3)	C19—C18	1.386 (5)
C11—H11A	0.9700	C1—H1A	0.9600
C11—H11B	0.9700	C1—H1B	0.9600
C10—H10A	0.9700	C1—H1C	0.9600
C10—H10B	0.9700	C16—H16	0.9300
C5—C8	1.456 (3)	C16—C17	1.356 (4)
C5—C4	1.402 (3)	C27—H27	0.9300
C5—C6	1.397 (3)	C27—C26	1.357 (5)
C8—H8	0.9300	C18—C17	1.377 (5)
C14—H14	0.9300	C18—C21	1.528 (5)
C14—C15	1.457 (3)	C25—H25	0.9300
C4—H4	0.9300	C25—C26	1.352 (5)
C4—C3	1.378 (3)	C26—H26	0.9300
C22—H22A	0.9700	C17—H17	0.9300
C22—H22B	0.9700	O2—C29	1.210 (3)
C22—C23	1.501 (3)	O3—H3	0.8200
C23—C28	1.379 (4)	O3—C29	1.320 (3)
C23—C24	1.388 (3)	C29—C30	1.478 (4)
C6—H6	0.9300	C30—H30A	0.9600
C6—C7	1.377 (3)	C30—H30B	0.9600
C15—C20	1.392 (4)	C30—H30C	0.9600
C15—C16	1.389 (3)	C21—H21A	0.9600
C2—C3	1.382 (4)	C21—H21B	0.9600
C2—C7	1.384 (3)	C21—H21C	0.9600
			
C11—N1—H1	108.1	C23—C28—C27	120.2 (3)
C11—N1—C22	113.02 (16)	C27—C28—H28	119.9
C10—N1—H1	108.1	C4—C3—C2	121.1 (2)
C10—N1—C11	110.26 (16)	C4—C3—H3A	119.4
C10—N1—C22	109.03 (14)	C2—C3—H3A	119.4
C22—N1—H1	108.1	C6—C7—C2	122.0 (2)
C13—C9—C10	118.82 (17)	C6—C7—H7	119.0
C8—C9—C13	117.84 (18)	C2—C7—H7	119.0
C8—C9—C10	123.32 (19)	C23—C24—H24	120.2
C9—C13—C12	117.95 (17)	C23—C24—C25	119.6 (3)
O1—C13—C9	121.50 (19)	C25—C24—H24	120.2
O1—C13—C12	120.50 (19)	C15—C20—H20	119.9
C13—C12—C11	118.64 (17)	C19—C20—C15	120.3 (3)
C14—C12—C13	118.29 (19)	C19—C20—H20	119.9
C14—C12—C11	123.07 (19)	C20—C19—H19	119.7
N1—C11—C12	109.89 (16)	C20—C19—C18	120.6 (3)
N1—C11—H11A	109.7	C18—C19—H19	119.7
N1—C11—H11B	109.7	C2—C1—H1A	109.5
C12—C11—H11A	109.7	C2—C1—H1B	109.5
C12—C11—H11B	109.7	C2—C1—H1C	109.5
H11A—C11—H11B	108.2	H1A—C1—H1B	109.5
N1—C10—C9	112.41 (15)	H1A—C1—H1C	109.5
N1—C10—H10A	109.1	H1B—C1—H1C	109.5
N1—C10—H10B	109.1	C15—C16—H16	119.4
C9—C10—H10A	109.1	C17—C16—C15	121.1 (3)
C9—C10—H10B	109.1	C17—C16—H16	119.4
H10A—C10—H10B	107.9	C28—C27—H27	119.7
C4—C5—C8	117.3 (2)	C26—C27—C28	120.5 (3)
C6—C5—C8	126.42 (19)	C26—C27—H27	119.7
C6—C5—C4	116.3 (2)	C19—C18—C21	118.7 (4)
C9—C8—C5	132.1 (2)	C17—C18—C19	118.4 (3)
C9—C8—H8	113.9	C17—C18—C21	122.8 (3)
C5—C8—H8	113.9	C24—C25—H25	119.7
C12—C14—H14	115.4	C26—C25—C24	120.7 (3)
C12—C14—C15	129.2 (2)	C26—C25—H25	119.7
C15—C14—H14	115.4	C27—C26—H26	119.9
C5—C4—H4	119.0	C25—C26—C27	120.2 (3)
C3—C4—C5	121.9 (2)	C25—C26—H26	119.9
C3—C4—H4	119.0	C16—C17—C18	121.4 (3)
N1—C22—H22A	108.6	C16—C17—H17	119.3
N1—C22—H22B	108.6	C18—C17—H17	119.3
H22A—C22—H22B	107.5	C29—O3—H3	109.5
C23—C22—N1	114.79 (16)	O2—C29—O3	121.5 (3)
C23—C22—H22A	108.6	O2—C29—C30	124.9 (3)
C23—C22—H22B	108.6	O3—C29—C30	113.5 (3)
C28—C23—C22	120.3 (2)	C29—C30—H30A	109.5
C28—C23—C24	118.8 (2)	C29—C30—H30B	109.5
C24—C23—C22	120.9 (2)	C29—C30—H30C	109.5
C5—C6—H6	119.4	H30A—C30—H30B	109.5
C7—C6—C5	121.1 (2)	H30A—C30—H30C	109.5
C7—C6—H6	119.4	H30B—C30—H30C	109.5
C20—C15—C14	122.6 (2)	C18—C21—H21A	109.5
C16—C15—C14	119.2 (2)	C18—C21—H21B	109.5
C16—C15—C20	118.1 (2)	C18—C21—H21C	109.5
C3—C2—C7	117.4 (2)	H21A—C21—H21B	109.5
C3—C2—C1	121.4 (2)	H21A—C21—H21C	109.5
C7—C2—C1	121.2 (2)	H21B—C21—H21C	109.5
C23—C28—H28	119.9		
			
N1—C22—C23—C28	−100.9 (2)	C14—C15—C20—C19	177.7 (2)
N1—C22—C23—C24	81.1 (3)	C14—C15—C16—C17	−179.5 (3)
C9—C13—C12—C11	−3.3 (3)	C4—C5—C8—C9	167.5 (2)
C9—C13—C12—C14	176.39 (18)	C4—C5—C6—C7	−3.6 (3)
O1—C13—C12—C11	179.07 (19)	C22—N1—C11—C12	−174.39 (16)
O1—C13—C12—C14	−1.3 (3)	C22—N1—C10—C9	177.83 (17)
C13—C9—C10—N1	20.4 (3)	C22—C23—C28—C27	−178.0 (2)
C13—C9—C8—C5	178.0 (2)	C22—C23—C24—C25	177.0 (2)
C13—C12—C11—N1	−32.5 (2)	C23—C28—C27—C26	0.6 (4)
C13—C12—C14—C15	−172.7 (2)	C23—C24—C25—C26	1.6 (5)
C12—C14—C15—C20	35.2 (4)	C6—C5—C8—C9	−14.1 (4)
C12—C14—C15—C16	−148.4 (3)	C6—C5—C4—C3	3.5 (3)
C11—N1—C10—C9	−57.5 (2)	C15—C20—C19—C18	0.5 (5)
C11—N1—C22—C23	60.2 (2)	C15—C16—C17—C18	2.9 (5)
C11—C12—C14—C15	7.0 (4)	C28—C23—C24—C25	−1.1 (4)
C10—N1—C11—C12	63.3 (2)	C28—C27—C26—C25	0.0 (5)
C10—N1—C22—C23	−176.78 (19)	C3—C2—C7—C6	2.7 (4)
C10—C9—C13—O1	−172.6 (2)	C7—C2—C3—C4	−2.8 (3)
C10—C9—C13—C12	9.8 (3)	C24—C23—C28—C27	0.0 (4)
C10—C9—C8—C5	−0.4 (4)	C24—C25—C26—C27	−1.1 (5)
C5—C4—C3—C2	−0.3 (4)	C20—C15—C16—C17	−2.9 (4)
C5—C6—C7—C2	0.6 (4)	C20—C19—C18—C17	−0.6 (5)
C8—C9—C13—O1	9.0 (3)	C20—C19—C18—C21	178.7 (3)
C8—C9—C13—C12	−168.70 (18)	C19—C18—C17—C16	−1.0 (5)
C8—C9—C10—N1	−161.29 (19)	C1—C2—C3—C4	177.3 (2)
C8—C5—C4—C3	−178.0 (2)	C1—C2—C7—C6	−177.4 (2)
C8—C5—C6—C7	178.0 (2)	C16—C15—C20—C19	1.2 (4)
C14—C12—C11—N1	147.8 (2)	C21—C18—C17—C16	179.6 (4)

### Hydrogen-bond geometry (Å, °)

**Table:**

Cg3 is the centroid of the C15–C20 ring.

**Table:**

*D*—H···*A*	*D*—H	H···*A*	*D*···*A*	*D*—H···*A*
N1—H1···Cl1	0.91	2.15	3.0490 (17)	171
O3—H3···Cl1	0.82	2.26	3.053 (2)	162
C11—H11A···Cl1^i^	0.97	2.72	3.602 (2)	151
C25—H25···Cg3^ii^	0.93	2.85	3.582 (4)	137
C30—H30C···Cg3^iii^	0.97	2.96	3.675 (4)	133

Symmetry codes: (i) *x*+1, *y*, *z*; (ii) −*x*+1, −*y*−1, −*z*; (iii) *x*, *y*−1, *z*.
